# Characteristics of patients with somatoform pain disorder

**DOI:** 10.1002/jgf2.735

**Published:** 2024-10-14

**Authors:** Lena Barth, Linda Baumbach, André Hajek, Lutz Götzmann, Katrin Feiks, Michael Rufer, Kyrill Schwegler, Bianca Schwennen, Kirstin Bernhardt, Uwe Wutzler, Paul Kaiser, Lutz Wittmann, Adrian Siegel

**Affiliations:** ^1^ Department of Psychology, MSH Medical School Hamburg Hamburg Germany; ^2^ Department of Health Economics and Health Services Research University Medical Center Hamburg‐Eppendorf Hamburg Germany; ^3^ Institute of Philosophy, Psychoanalysis and Cultural Studies (IPPK) Berlin Germany; ^4^ Clinic of Psychosomatics, Psychiatry and Psychotherapy University of Lübeck Lübeck Germany; ^5^ Department of Psychosomatic Medicine and Psychotherapy, Segeberger Kliniken Bad Segeberg Germany; ^6^ Department of Psychiatry University Hospital of Zuerich Zürich Switzerland; ^7^ Center for Psychiatry and Psychotherapy, Hospital Zugersee Triaplus AG Zug Switzerland; ^8^ Department of Psychosomatic Medicine Inselspital Bern Switzerland; ^9^ Klinik für Psychosomatische Medizin Und Psychotherapie MediClin Seepark Klinik Bad Bodenteich Germany; ^10^ Blomenburg Privatklinik Selent Germany; ^11^ Asklepios Fachklinikum Stadtroda, Psychotherapie Und Psychosomatik Stadtroda Germany; ^12^ International Psychoanalytic University Berlin Germany; ^13^ Neurozentrum Thalwil Zürich Switzerland

**Keywords:** chronic pain, functional somatic symptoms, psychotherapy, quality of life, somatoform pain

## Abstract

**Background:**

Patients with somatoform pain experience physical pain that cannot be attributed to any underlying medical or physiological cause, and it is often thought to be related to psychological factors. Health professionals encounter difficulties identifying this specific type of chronic pain, leading to suboptimal treatment strategies. Therefore, we aimed to describe the characteristics of patients with somatoform pain, to support the identification of affected patients.

**Methods:**

We collected and analyzed a cross‐sectional survey data from 200 patients with somatoform pain admitted to one of three psychosomatic centers in Germany between August 2013 and July 2014. The survey contains 10 different categories, all of them referring to pain‐related topics. Within the survey, we analyzed validated as well as non‐validated questionnaires. Here, we present the following five: Personal data, Body: Pain perception, Cognition: Pain processing, Pain behavior, and Physical complaints.

**Results:**

Our results highlight that most patients with somatoform pain experience it in several body parts and as persisting, lasting >12 h/day (50%), and constantly changing (71%). Furthermore, patients indicate feelings of helplessness, by agreeing to the expressions the pain controls me (70%). Finally, we found that pain is predominantly seen as suffering, failing to convey emotional pain, despite cognitively acknowledging the dependency of emotional and physical pain.

**Conclusion:**

The study identified specific and distinctive characteristics in the emotional and behavioral responses of patients with somatoform pain, potentially distinguishing them from other patients with chronic pain.

## Abstract 2

The study analyzed survey data from 200 patients with somatoform pain, finding that most experience persistent pain in multiple body parts, feel a sense of helplessness, and predominantly perceive pain as suffering rather than emotional distress, highlighting distinctive emotional and behavioral responses in this group.

## 1 INTRODUCTION

According to the International Association for the Study of Pain (IASP), pain is defined as an unpleasant sensory and emotional experience associated with actual or potential tissue damage.^1^ Medical classifications such as the International Classification of Diseases (ICD) and the Diagnostic and Statistical Manual of Mental Disorders (DSM) delineate various concepts related to “somatoform pain.” In ICD‐10, somatoform pain disorder (F45.40) is characterized by pain lacking a discernible physical basis. Further, since 2009, the German ICD‐10 contains an additional subtype, i.e. chronic pain disorder with somatic and psychological factors (F45.41). This diagnosis addresses the frequent interplay between somatic and psychological factors. DSM V focuses on the impact of pain on mental states rather than vice versa. Conversely, the upcoming ICD‐11 introduces the concept of “bodily distress disorder (BDD),” which emphasizes a bidirectional relationship between bodily and emotional states, suggesting that emotional distress both triggers and sustains somatoform pain^2^, ^3^ (International Advisory Group for the Revision of ICD‐10, 2010). Despite the recognition of pain as a biopsychosocial phenomenon, psychosocial aspects often receive inadequate attention.^4^ Difficulties in emotional regulation, heightened negative emotions, interpersonal challenges, and adverse childhood experiences are frequently observed in individuals with somatoform pain.^5^, ^6^, ^7^, ^8^, ^9^ Neurobiological research indicates shared neural networks between interpersonal distress and physical pain. To underline a biographical component, adverse childhood experiences are strongly correlated with somatoform pain disorders, often manifesting as unfulfilled desires for closeness and fear of rejection.^10^, ^11^, ^12^ Contemporary biopsychosocial models advocate for a multidimensional conceptualization of somatoform pain, addressing both central and peripheral aspects of heightened pain perception. However, systematic assessment tools for psychosocial aspects remain limited, hindering diagnostic processes as well as the development of effective treatment strategies that integrate psychological therapies capable of addressing such factors.^13^, ^14^

Existing instruments for assessing somatoform pain often focus either on physical or psychological dimensions, with few accounting for the complex interplay between physical, psychological, and social factors.^15^, ^16^, ^17^, ^18^ According to Ringel et al.,^16^ handling patients with somatoform disorders requires a balanced approach. Positive actions include actively validating patients through thorough questioning and ensuring comprehension, offering reassurance, conducting brief physical examinations focusing on discomfort areas, and delicately introducing psychosocial aspects in everyday language. Regular, time‐based medical appointments should be established. Negative interventions involve unnecessary diagnostic procedures or invasive treatments, using language that downplays the situation, suggesting unverified causal hypotheses, or even stigmatizing patients. It is essential not to solely attribute symptoms to psychosocial factors, avoiding the “psychological” label. A holistic, empathetic, and patient‐centered approach is crucial for effectively managing somatoform patients.

Differentiating somatoform pain disorder from other chronic pain conditions presents significant diagnostic challenges due to the substantial overlap in symptoms, lack of specific biomarkers, and the complex interaction of psychological and cultural factors. For instance, symptoms such as persistent pain and fatigue are common across various chronic pain disorders, complicating the identification of somatoform pain.^7^ Additionally, the absence of objective diagnostic markers further complicates accurate diagnosis, often leading to potential misdiagnosis or inadequate treatment. The role of psychological and cultural influences on pain perception adds another layer of complexity, requiring clinicians to navigate these factors carefully.^19^

To date, health care professionals, besides psychotherapists, struggle to identify somatoform pain patients and differentiate them from other chronic pain patients. Consequently, they often provide inadequate treatments,^1^ which leads to a vicious circle of misunderstanding and frustration on the patient as well as the health professional's side. To support the identification of patients with somatoform pain, which assists an efficient doctor–patient interaction, we aim to describe the characteristics of this specific patient group.

## 2 METHODS

### 2.1 Study design

This publication is a survey‐based multicenter cross‐sectional study aiming at the description of patients with a somatoform pain disorder. In reporting the results, we follow the Strengthening the Reporting of Observational Studies in Epidemiology (STROBE) guidelines.^20^ This study was approved by the responsible ethics committees at the University of Lübeck, Germany on 13.08.2013, ID: 13–08.

### 2.2 Setting and data source

The cross‐sectional study comprised 200 patients admitted consecutively to the Clinic for Psychosomatic Medicine and Psychotherapy of the University Hospital Kiel, the Segeberger Klinik, and the Asklepios‐Fachklinikum Stadtroda, Germany, with a diagnosis of somatoform pain disorder (F45.40). In Germany, these patients are typically referred to by their general practitioner to such an inpatient treatment. The data were collected consecutively from 200 patients admitted to one of the centers between August 2013 and July 2014. The patients were informed about the study and asked if they would like to participate. If they agreed and signed a related consent form, they were included in the study. The response rate was 100%. Most participants spontaneously completed the survey; however if not so, they were asked to supplement questionnaires the following days. This led to a complete dataset with no missing values.

### 2.3 Participants

All patients included in this study needed to have a diagnosis of somatoform pain disorder (ICD‐10: F45.40), be at least 18 years old and have good German language skills. This ICD‐10 diagnosis was made, based on the admission interview, using the ICD‐10 criteria: predominant complaint is a persistent, severe, and distressing pain that cannot be adequately explained by a physiological process or physical disorder. It occurs in association with emotional conflicts or psychosocial stressors, which play a central role in the onset, severity, exacerbation, or maintenance of the pain. Patients were excluded if they had a physical brain condition or a diagnosis of a psychotic or eating disorder. Diagnosis was made by an experienced clinician, based on the admission interview. This clinical diagnosis was verified with the Structured Clinical Interview for DSM‐IV.^21^ Although the diagnosis of somatoform pain disorder has been abandoned in DSM‐5 and replaced by the broader concept of somatic symptom disorder, we still used somatoform pain disorder for the purpose of this study, also because the use of somatic symptom disorder for patients with chronic pain is not recommended.^22^ Consequently, all included patients fulfill the ICD‐10 criteria for the diagnosis of somatoform pain: The ICD‐10 criteria for somatoform pain disorder require the presence of persistent, severe pain that is excessively preoccupying and distressing, with psychological factors playing a significant role, and with no alternative physiological or psychiatric explanation for the pain.

### 2.4 Survey

The survey was organized into 10 main categories: Personal data, Body: Pain perception, Cognition: pain processing, Emotions: satisfaction, mood, stress, Pain behavior, Contacts: doctor's appointments, communication, support, Worries and apprehension, Physical complaints, Mood during the last week, and State of health and quality of life. The last four categories contain each a validated questionnaire while the former categories include information, typically obtained during psychotherapeutic treatments. In this article, we focus and present the main categories: Personal data, Body: Pain perception, Cognition: pain processing, Pain behavior, and Physical complaints. Detailed information on the involved questions is provided later. Further, a brief summary of the remaining categories is presented.

*Personal data* contains information on 11 items: age (in years), gender (female, male), height (cm), profession (free text), current activity (free text), labor status (full time, part time, unemployed), Labor capacity (capable of work, incapable), intake of current medication (free text) and pain duration (in years). In this paper, we dichotomised the variable current pain medication into yes, no. The other two variables with a free text answer option are not presented in this paper.

#### 2.4.1 Body

*Pain perception* covers the information of seven main questions with one to six subheadings. The first main question was “Where do you have pain?” participants could indicate the presence or absence of pain individually for the subheading head, stomach, back, and extremities. Second, information on the quality of pain was obtained. Participants indicated the presence or absence of the following pain qualities: dull/pressing, sharp/stabbing, pounding/throbbing, dragging/ripping, and burning. Afterwards, they were asked if the pain was radiating (yes/no). The fourth question was on the maximal pain level during the last 4 weeks on a scale from 0 – no pain to 10 – worst pain. Participants could choose between 0–1, 2–3, 4–5, 6–7, 8–9 and 10. Furthermore, the participants indicated if the pain was worst: during the morning (yes, no), noon (yes, no), evening (yes, no), night (yes, no), constantly changing (yes, no), and always the same (yes, no). In the sixth question, participants indicate the normal duration of their daily pain as less than 2 h (yes, no), between 2 and 4 h (yes, no), between 4 and 8 h (yes, no), between 8 and 12 h (yes, no), and more than 12 h (yes, no). Finally, the participants were asked if the pain was worse at rest (yes, no), during loading (yes, no), changing – once at rest than during loading (yes, no), always the same – at rest and at loading (yes, no).

#### 2.4.2 Cognition

*Pain processing* involves four main items with 3–13 subheadings. The participant could indicate totally agree, agree, disagree, or completely disagree to each item and subheading. In this article, we dichotomized the responses into strongly agree and agree vs. disagree and completely disagree. In the first item, the participant was asked about the origin of their pain. They rated the pain into three subheadings: (1) constitutional, (2) psychological, and (3) constitutional as well as psychological. In the second item, participants indicated if their pain meant: suffering, punishment, injustice, warning, relief, chance, fate, and pleasure. In the third item, participants were asked regarding their attitude toward their body. They evaluated the subheadings: In general, I like my body, I accept my body, I trust my body, I am disappointed by my body, sometimes I would like to have a different body, sometimes I hate my body. Finally, participants were asked to rate the following general statements about pain: Pain subserve self‐discovery, pain dependence on the culture, pain depends on faith, pain is superfluous, pain needs to combated, pain is vital, pain can facilitate a conscious life, pain is an independent disease, psychological pain is worse than constitutional, constitutional pain is worse than psychological, there is no difference between constitutional and psychological pain, constitutional and psychological pain interact with each other, in the population there are prejudices toward psychological diseases.

*Pain behavior* contains six questions with 5–12 subheadings. First participants were asked if there are situations in which the intensity of the pain is changing for each subheading they could indicate total agreement, agreement, disagreement, or complete disagreement. The following five subheadings were rated: When I am relaxed, the pain is less intense, when I am distracted, the pain is less intense, my pain increases when I am upset, my pain increases when I get angry, my pain increases when I am frustrated. The next item is the ability to reduce pain by movements, relaxing, distraction, pleasure, medication, and conversations. The ability to reduce the pain of each of these six subheadings was rated by the participant as very strong, strong, weak, or not at all. Next participants evaluated areas in which they feel very restricted, restricted, less, restricted, or not restricted by the pain. The areas in the subheading involved: self‐supply (e.g., groceries), relationships (family, partner, and friends), profession/labor, independence, decisions, physical activity, eating/drinking, sleep, and sexuality. As part of the fourth item, participants were asked to imagine they would have no pain from tomorrow on, then they should indicate how they expect to react. They rated the following six subheadings as strongly agree, agree, disagree, and completely disagree: I would be relieved and satisfied, I would exuberantly celebrate, I would disbelief and be skeptical, I would solve other problems, I would not change anything, and I can not imagine this situation (of freedom of pain). In the following Item, participants were asked how they can influence their pain. They rated the following 10 statements again on a four‐point‐scale from strongly agree to completely disagree: I can influence the pain via my body, I can influence my pain through my behavior, I can not control my pain, the pain controls me, I can influence the pain, the pain is influencing me, and I could not accept if the pain would last forever. For the yet five mentioned pain behavior items, we again dichotomised the response options by merging the upper two and lower two response options. In the final sixth item in this category, the participants were asked what they expected from a therapy and what their goals would be. They evaluated the following 12 subheadings as being a goal (yes, no): freedom of pain, reduction of pain, learning a better way to handle the pain, understanding the cause of the pain, reducing the need for pain medication, obtaining knowledge on pain‐keeping and increasing factors, learning to arrange with the pain, increasing the self‐confidence, increasing the professional workability for the job, increasing the body's flexibility, regaining zest for life/vital energy, nothing.

*Physical complaints* were measured with the Screening for Somatoform Disorders (SOMS‐2) questionnaire. SOMS‐2 is a widely used screening tool for somatoform disorders. Respondents were asked to indicate whether they suffered from 47 different somatic symptoms (e.g., headache, back pain, nausea), for which no organic cause has been identified by a physician, during the past 2 years. The number of endorsed symptoms is condensed into the somatising complaint index which was used to indicate the severity of somatic symptoms in this study. A cut‐off of ≥17 has been suggested to indicate somatoform problems.^18^ The internal consistency of the 47 symptom items in the present sample was ⍺ = 0.92.

Of the remaining categories: Affects: your satisfaction, mood, stress, Contacts: doctors appointments, communication, support, Worries and apprehension, Mood during the last week, and State of health and quality of life, the latter three based on validated questionnaire, the STAI,^23^ the BDI,^24^ and the SF‐12,^18^ respectively. The category on affects involved eight questions with 4–31 subheadings and the category on contacts involved three questions with 12–15 subheadings.

### 2.5 Statistical methods

We analyzed the data utilizing SPSS and provided descriptive statistics as mean and standard deviations (SD) or frequencies with proportions [*n* (%)], respectively.

## 3 RESULTS

All characteristics of the patients are presented in Table 1. Their mean age was 49 years and 80% were female. Sixty‐three percent of the sample indicated to be unable to work.

The pain was experienced as persistent >12 h/day and with high‐intensities (>7) by 50% and 58% of the patients, respectively. Patients reported the intensity to be constantly changing throughout the day (71%) and mostly as pulling (72%) and dull (63%). The vast majority of patients reported pain in the back (89%) and in extremities (86%), with 59% of all patients reporting more than two different pain locations.

Patients saw the reason for the pain as being constitutional (73%), psychological (69%) and both (87%). They also agreed to physical and emotional pain to be depending on each other (92%). For most patients, the pain meant suffering (85%) and warning (75.5%). Finally, they indicated that the population has prejudiced against patients with psychological diseases (97%).

The pain significantly disrupted the patient's daily lives as indicated by more than 50% agreeing to each suggested area to be limited by the pain, involving work (90.5%), physical activities (83%), and sleep (78.5%). Patients indicated that they feel helpless by agreeing to the statements: “*I cannot control the pain”* (66%), *“The pain controls me”* (70%), and *“The pain influences me”* (87%). To reduce the pain, mostly medication was mentioned by the patients (57%), followed by relaxation (47%) and distraction (37%). Only 18% consider conversations to allow a pain reduction. The patient's strong desire to confront and manage their pain was evident by agreeing to several of the suggested therapy goals. However, 55% could not imagine being pain‐free.

**TABLE 1:** Characteristics of the included participants.

(A) Personal data	
Age, mean (SD)	49.1 (9.9)
Pain duration, mean (SD)	12.5 (11.8)
Sex, *n* (%)	
• Female	160 (80)
• Male	40 (20)
Labour situation, *n* (%)	
• Full time employment	74 (37)
• Part time employment	46 (23)
• Without work	58 (29)
Work ability, *n* (%)	
• Able to work	51 (25.5)
• Unable to work	126 (63)
Intake of pain medication, *n* (%)	
• Yes	154 (77)
• No	43 (21.4)
(B) Body: pain perception	
Pain in the following body parts, *n* (%).	
• Head	128 (64)
• Stomach	68 (34)
• Back	178 (89)
• Extremities	171 (85,5)
Pain quality, *n* (%)	
• Dull	127 (63.5)
• Sharp/stabbing	115 (57.5)
• Throbbing	81 (40.5)
• Pulling	144 (72)
• Burning	96 (48)
Radiation of pain, *n* (%)	175 (87.5)
Pain intensity on a scale from 0 no to 10 high pain, *n* (%)	
• 0–1	0 (0)
• 2–3	4 (2)
• 4–5	17 (8.5)
• 6–7	63 (31.5)
• 8–9	93 (46.5)
• 10	23 (11.5)
When is the pain the worst, *n* (%)	
Ín the…	
• Morning	82 (41)
• Noon	38 (19)
• Evenings	78 (39)
• Nights	50 (25)
• Constantly changing	142 (71)
• Always the same	34 (17)
Normal duration of pain in hours, *n* (%)	
• <2 h	17 (8.5)
• 2–4 h	37 (18.5)
• 4–8 h	50 (25)
• 8–12 h	42 (21)
• >12 h	100 (50)
When is the pain the worst, *n* (%)?	
• At rest	43 (21.5)
• Under stress	92 (46)
• Changing	99 (49.5)
• Independent of resting and loading	57 (28.5)
(C) Cognition: pain processing	
What is the reason for the pain, *n* (%)	
The pain is…. (agree)	
• Constitutional	145 (72.5)
• Psychological	137 (68.5)
• Both constitutional and psychological	174 (87)
Personal reason of pain, *n* (%)	
The pain means to me… (agree)	
• Suffering	170 (85)
• Punishment	42 (21)
• Injustice	50 (25)
• Warning	151 (75.7)
• Relief	13 (6.5)
• Chance	24 (12)
• Fate	71 (35.5)
• Desire/Pleasure	19 (9.5)
Attitude toward the body, *n* (%)	
Agreement on	
• In general, I like my body	118 (59)
• I accept my body	139 (69.5)
• I trust my body	110 (55)
• I am disappointed from my body	73 (36.5)
• Sometimes I would like to have a different body	112 (56)
• Sometimes I hate my body	69 (34.5)
• Pain serves self‐discovery	49 (24.5)
• Pain is dependent on culture	25 (12.5)
• Pain is dependent on believing	13 (6.5)
• Pain is superfluous	60 (30)
• Pain needs to be combated	161 (80.5)
• Pain is vital	118 (59)
• Pain can support conscious life	143 (71.5)
• Pain is an independent disease	93 (46.5)
• Emotional pain is worse than physical pain	90 (45)
• Physical pain is worse than emotional pain	64 (32)
• There is no difference between emotional and physical pain	86 (43)
• Physical and emotional pain depend each other	184 (92)
• The population has prejudiced against patients with psychological diseases	194 (97)
(D) Pain behavior	
Situations in which the strength or intensity of your pain changes (agree)	
• When I relax, my pain becomes less intense	125 (62.5)
• When I distract myself, my pain becomes less intense	118 (59)
• My pain increases when I get upset	138 (69)
• My pain increases when I get angry	133 (66.5)
• My pain increases when I am frustrated	124 (62)
Pain can be reduced by… (agree)	
• Movement	47 (23.5)
• Relaxation	94 (47)
• Distraction	74 (37)
• Enjoyment	23 (11.5)
• Medications	114 (57)
• Conversations	37 (18.5)
In which areas do you feel limited by the pain? (agree)	
• Self‐care (e.g., going shopping)	110 (55)
• Relationships (family, partnership, friends)	141 (70.5)
• Occupation / Work	181 (90.5)
• Independence	126 (63)
• Decision‐making	105 (52.5)
• Physical movement	166 (83)
• Eating/Drinking	43 (21.5)
• Sleep	157 (78.5)
• Sexuality	129 (64.5)
Imagine that starting tomorrow, you would have no pain at all. How do you think you would react (agree)?	
• I would be relieved and satisfied	196 (98)
• I would celebrate exuberantly	78 (39)
• I would be incredulous and skeptical	122 (61)
• I could then solve other problems	137 (68.5)
• Nothing would change	19 (9.5)
• I cannot imagine such a situation (pain‐free)	109 (54.5)
In what ways can your pain be influenced (agree)?	
• I can influence the pain through my body	52 (26)
• I can influence the pain through my thoughts	47 (23.5)
• I can influence the pain through my emotions	60 (30)
• I can influence the pain through my behavior	103 (51.5)
• I cannot control my pain	132 (66)
• I can control my pain	25 (12.5)
• The pain controls me	140 (70)
• I can influence the pain	59 (29.5)
• The pain influences me	173 (86.5)
• I could never accept if my pain were to last forever	123 (61.5)
What would you expect from therapy, i.e., what goals would you set for yourself in therapy (agree)?	
• Freedom from pain	119 (59.5)
• Relief from pain	187 (93.5)
• Learn to manage pain better	185 (92.5)
• Better understand the causes of pain	191 (95.5)
• Reduce the need for pain medication	171 (85.5)
• Gain knowledge about pain‐aggravating/maintaining factors	168 (84)
• Learn to cope with the pain	144 (72)
• Strengthen self‐confidence	166 (83)
• Improve performance at work	174 (87)
• Enhance physical mobility	185 (92.5)
• Regain enthusiasm for life	185 (92.5)
• Nothing	1 (0.5)

## 4 DISCUSSION

We provide an overview of the characteristics of 200 patients with somatoform pain to allow identification and adequate treatment of these patients. Our results highlight somatoform pain persistent (>12 h/day) and constantly changing nature, which is accompanied by feelings of helplessness among patients. A passive assistance‐seeking tendency and reliance on medical interventions might be prominent and combined with neglecting a potential psychological cause of pain. Although somatoform pain patients desire relief and satisfaction through reduced pain, envisioning such relief remains challenging.

Notably, we found that pain is predominantly seen as suffering, failing to convey emotional pain, despite cognitively acknowledging the dependency of emotional and physical pain. The disconnect between pain and emotions persists, as patients do not recognize that conversations allow the investigation of relationships between the two. In treating somatoform disorder patients, it is vital to consider childhood attachment issues and their struggle to differentiate physical pain from emotional distress.

Therapy focuses on communication to uncover the interpersonal significance of symptoms and access the patient's inner world, enabling emotional expression and differentiation between physical symptoms and emotions. Additionally, it addresses early relationship experiences to replace insecure attachment patterns with secure ones, as suggested by Egle and Zentgraf.^25^

Ringel^16^ addresses challenges in medical as well as therapeutic interventions with somatoform patients. He identified an implicit dialogue between doctors and their patients causing a tremendous amount of distress in both parties. Our data provide similar findings: patients exhibit a high level of expectation toward the healthcare system and seek support due to potential pragmatic, organic causes, as they perceive fewer opportunities to take action themselves highlighted by their agreement to the statements “I cannot control the pain” and “Pain can be reduced by medication.” This vicious circle might repeat adverse childhood experiences, associated with detrimental effects on both somatic and mental health aspects.

Individuals who were maltreated in childhood have a higher risk for morbidity and mortality related to chronic diseases as well as neurobiological, endocrine, and immunological changes.^25^ In this context, the hypothesis can be formulated that the experienced helplessness (“I cannot control my pain”) and associated passivity (“The pain influences me”) reflect an early learned helplessness and thus are being re‐enacted. This leaves patients more susceptible to heightened physical and emotional reactions, coupled with reduced self‐regulation. Hence, patients with somatoform pain still experience emotional distress during physical interactions with others. Further, it might be hypothesized that a patient's approach to relationships combines a desire for them (“the pain limits my relationships”) with a fear of rejection (“The population has prejudiced against patients with psychological diseases”), often hindering their engagement in interactions. Psychotherapists often have more time with the patient and education on emotional management strategies, nonetheless, knowledge about these specific circles of interaction is vital for other health professionals as well.

In summary, our data might assist in accurately identifying somatoform pain patients with the following aspects: In cases of somatoform pain disorder, the pain manifests as a persistent, diffuse sensation that proves challenging to discern or attribute to a specific source. Patients acknowledge the dependency of emotional and psychological pain but do not translate this to themselves and seem not to consider any emotional factors underlying their discomfort. This information might assist clinicians in distinguishing somatoform patients from patients with medically unexplained symptoms or other chronic pain diseases. For example, in fibromyalgia, patients often recognize their pain as a physical condition, but there might be a greater acceptance of the interplay between their pain and psychological factors, such as stress and mood disorders.^26^ Furthermore, there tend to be discrepancies among patients regarding the causes, potential remedies, and necessary behaviors in relation to their experience of pain (e.g., desire to confront and manage pain, yet the feeling of limitation in controlling its intensity, identifying a strong dependency solely on medication for relief), further complicating the understanding and treatment of this condition.

Consequently, in case, health professionals identify a potential patient with somatoform pain the following suggestions, in line with Sauer & Eich,^1^ might be followed: validate patients, offer reassurance, conduct short physical examinations, slowly introduce psychosocial aspects as well as regular appointments. This might be more effective than keeping the focus on finding a physiological cause of the pain as well as focusing on psychological reasons, before patients are ready. Finally, in line with the S3 guidelines psychotherapeutic care should be initiated. Similar findings as well as the efficacy of a psychotherapeutic intervention (both psychodynamic psychotherapy and behavioral therapy) was additionally analyzed through a systematic review by Sauer & Eich.^1^

## 5 STRENGTHS AND LIMITATIONS

In this initial paper, we offer a comprehensive description of our patient cohort, utilizing tools that, while partially validated, are the only viable options available. Notably, our patient sample primarily comprises hospitalized individuals, potentially introducing bias compared to and the general population but also to those with somatoform pain visiting general practitioners. While we assume that the latter is to some degree similar in its patient characteristics, this needs empirical validation. Further, the sample only includes German citizens. It would be an interesting future research to compare cultural differences in the perception and the management of pain as well as the associated medical interventions. For example, studies have shown that individuals from Asian cultures might underreport pain due to cultural norms that value stoicism and endurance, whereas individuals from Western cultures might be more likely to express pain and seek relief.^24^, ^27^ Our study's strengths lie in its detailed patient description, but it is crucial to acknowledge limitations, including tool validity and potential sampling bias, to ensure the robustness and applicability of our findings. Addressing these limitations and further investigating temporal dynamics is imperative for future research in this domain.

## 6 CONCLUSION AND FUTURE RESEARCH

The study examines 200 patients with somatoform pain and identifies key characteristics, including the persistent and constantly changing nature of the pain, patients' feelings of helplessness, and a tendency to rely on medical interventions while neglecting potential psychological causes. It also highlights the disconnect between pain and emotions in these patients, which can emphasize the importance of addressing childhood attachment issues and improving communication in therapy. In line with clinical guidelines, we suggest that health professionals should focus on validation, reassurance, and psychosocial aspects in treatment rather than solely searching for a physiological cause of the pain. Further research needs to carefully address this specific finding and compare it to populations with other chronic pain disorders as well as a healthy population in order to verify our results as discriminative.

## AUTHOR CONTRIBUTIONS

**Lena Barth:** Conceptualization of the manuscript, Methodology of the manuscript, Formal Analysis, Writing – Original Draft, Writing – Review & Editing. **Linda Baumbach:** Conceptualization of the manuscript, Methodology of the manuscript, Formal Analysis, Writing – Original Draft, Writing – Review & Editing. **André Hajek:** Conceptualization of the manuscript, Methodology of the manuscript, Formal Analysis, Writing – Review & Editing. **Lutz Götzmann:** Conceptualization of the study, Methodology of the study, Data collection, Writing – Review & Editing. **Katrin Feiks**: Conceptualization of the study, Data Collection, Review & Editing. **Michael Rufer:** Conceptualization of the study, Data Collection, Review & Editing. **Kyrill Schwegler:** Conceptualization of the study, Data Collection, Review & Editing. **Bianca Schwennen:** Conceptualization of the study, Data Collection, Review & Editing. **Kirstin Bernhardt:** Conceptualization of the study, Data Collection, Review & Editing. **Uwe Wutzler:** Conceptualization of the study, Data Collection, Review & Editing. **Paul Kaiser:** Methodology of the study, Writing – Review & Editing. **Lutz Wittmann:** Methodology of the study, Writing – Review & Editing. **Adrian Siegel:** Conceptualization of the study, Methodology of the study, Data collection, Writing – Review & Editing.

## CONFLICT OF INTEREST STATEMENT

The authors have stated explicitly that there are no conflicts of interest in connection with this article.

## ETHICS APPROVAL STATEMENT

The study was approved by the responsible ethics committees at the University of Lübeck on 13.08.2013, ID: 13‐08.

## PATIENT CONSENT STATEMENT

All participants provided written informed consent.

## Data Availability

The data are available for sharing upon reasonable request.
